# DEAD-box RNA helicase 21 interacts with porcine circovirus type 2 Cap protein and facilitates viral replication

**DOI:** 10.3389/fmicb.2024.1298106

**Published:** 2024-02-06

**Authors:** Jianwei Zhou, Jie Zhao, Haoyu Sun, Beining Dai, Ning Zhu, Qianhong Dai, Yonghui Qiu, Dedong Wang, Yongqiu Cui, Jinshuo Guo, Xufei Feng, Lei Hou, Jue Liu

**Affiliations:** ^1^College of Veterinary Medicine, Yangzhou University, Yangzhou, China; ^2^Jiangsu Co-innovation Center for Prevention and Control of Important Animal Infectious Diseases and Zoonoses, Yangzhou University, Yangzhou, China

**Keywords:** porcine circovirus type 2, capsid protein, nuclear localization signal, DEAD-box RNA helicase 21, virus replication

## Abstract

Porcine circovirus type 2 (PCV2) is the etiological agent of PCV2-associated diseases that pose a serious threat to the swine industry. PCV2 capsid (Cap) protein has been shown to interact with DEAD-box RNA helicase 21 (DDX21), an important protein that regulates RNA virus replication. However, whether the interaction between DDX21 and the PCV2 Cap regulates PCV2 replication remains unclear. Herein, by using western blotting, interaction assays, and knockdown analysis, we found that PCV2 infection induced the cytoplasmic relocation of DDX21 from the nucleolus in cultured PK-15 cells. Moreover, the nuclear localization signal (NLS) of PCV2 Cap interacted directly with DDX21. The NLS of PCV2 Cap and ^763^GSRSNRFQNK^772^ residues at the C-terminal domain (CTD) of DDX21 were essential for the dual interaction. Upon shRNA-mediated *DDX21* depletion in PK-15 cells, we observed impaired PCV2 replication via a lentivirus-delivered system, as evidenced by decreased levels of viral protein expression and virus production. In contrast, the replication of PCV2 increased in transiently DDX21-overexpressing cells. Our results indicate that DDX21 interacts with PCV2 Cap and plays a crucial role in virus replication. These results provide a reference for developing novel potential targets for prevention and control of PCV2 infection.

## Introduction

The circular replication-associated protein (Rep)-encoding single stranded (CRESS) DNA virus emergence in diverse host has been associated with severe disease (Zhao et al., 2019). The CRESS DNA viruses have seven family members: *Circoviridae*, *Nanoviridae*, *Smacoviridae*, *Genomoviridae*, *Bacilladnaviridae*, *Geminiviridae*, and *Redondoviridae*, established by the International Committee on the Taxonomy of Viruses (ICTV) (Zhao et al., 2019; Abbas et al., 2021). Porcine circovirus (PCV) belongs to the genus *Circovirus*, family *Circoviridae*. The genus *Circovirus* has been discovered in different animal species, including pigs, ducks, dogs, minks, rats, palm civets, geese, pigeons, canaries, and parrots (Schoemaker et al., 2000; Phenix et al., 2001; Todd et al., 2001; Lorincz et al., 2011; Nauwynck et al., 2012; Li et al., 2013; Lian et al., 2014). PCV is a non-enveloped, circular, single-stranded DNA virus with a 1.7–2.0-kb genome (Breitbart et al., 2017). PCV is the smallest known virus capable of autonomous replication in mammalian cells and is classified into four genotypes: PCV1, PCV2, PCV3, and PCV4 (Tischer et al., 1982; Allan et al., 1998; Palinski et al., 2017; Zhang et al., 2020). PCV2 is the major etiological agent of porcine circovirus-associated diseases (PCVAD), and porcine circovirus-like virus is also associated with PCVAD (Liu et al., 2021). Six PCV2-encoded viral proteins have been identified over the past decades (Mankertz et al., 1998; Nawagitgul et al., 2000; He et al., 2013; Lv et al., 2015; Li et al., 2018). The capsid protein acts as the major packaging protein of the PCV genome, regulating genome replication by binding to the Rep protein. The PCV Cap is also a major host-protective immunogen and essential for viral replication (Finsterbusch et al., 2009; Wiederkehr et al., 2009). Therefore, a comprehensive understanding of the functions of Cap can provide insights into the viral replication cycle and knowledge of therapeutic and prophylactic interventions for infections with PCV2.

The replication of circovirus is assumed to occur though a rolling circle replication (RCR) mechanism. Circovirus does not encode DNA polymerase, and hence, the host cellular replication machinery is required for *de novo* DNA synthesis (Heath et al., 2006). As for all PCVs, the conserved N-terminus of Cap protein comprise a nuclear localization signal (NLS) (Zhou et al., 2021a,b). The amino acid sequences of PCV2 Cap are extremely different from those of PCV1, PCV3, or PCV4 Cap, but their motifs are highly similar within the NLSs in spite of different PCV genotypes (Zhou et al., 2021a,2022a). The NLSs are critical elements of virus-encoding proteins (Matthews, 2001; Wurm et al., 2001). Some virus-encoding proteins are considered important in virus transcription and translation, cell cycle, and division (Salvetti and Greco, 2014). Other NLS-comprising proteins target the nucleus to change cell morphology (Hiscox, 2002). Thus, mapping the host cellular proteins binding to PCV2 Cap comprising an NLS will help understand the replication of PCV2 infection.

PCV2 Cap interacted with DEAD-box RNA helicase 21 (DDX21) in a proteomic study (Zhou et al., 2020, 2022a). DDX21 is considered as a plenteous nucleolar protein that connects with ribosomal RNA (rRNA) and small nucleolar RNAs (snoRNAs) to facilitate RNA metabolism (Valdez et al., 1996; Calo et al., 2015). DDX21 comprises a helicase domain that bears the DEXD sequence along with the flanking N-terminal and C-terminal domains and is proposed to interact with multiple cellular proteins (Fuller-Pace, 2006; Ali, 2021). It has been found to regulate RNA virus replication. For example, DDX21 inhibits the replication of influenza A virus by decreasing polymerase activity and reducing virus replication via interaction with PB1 protein (Chen et al., 2014). Dengue virus infection caused the cytoplasmic redistribution of DDX21 to suppress viral replication (Dong et al., 2016). Similarly, DDX21 regulates the replication of Borna disease virus by modulating mRNA translation (Watanabe et al., 2009). In addition, it acts as a cellular restriction factor of FMDV IRES-dependent translation and replication (Abdullah et al., 2021).

In addition, DDX21 also regulates host cellular innate immune responses to viruses (Bonaventure and Goujon, 2022; Ullah et al., 2022). For example, DDX1, DDX21, and DHX36 regulate host innate immune responses (Li et al., 2022a). Besides, cleavage of DDX21 suppresses host immune responses (Wu et al., 2021). Nevertheless, all studies on the roles of DDX21 in viral replication has only been investigated on RNA viruses, and the study on contribution of DDX21 to DNA virus replication was explored once (Hao et al., 2019). Therefore, it is essential to demonstrate whether binding of DDX21 to Cap regulates the life cycle of PCV2.

In the present study, our results demonstrate that DDX21 is transported from the nucleolus to the cytoplasm during PCV2 infection. The NLS of the PCV2 Cap and ^763^GSRSNRFQNK^772^ residues at the CTD of DDX21 are essential for the dual interaction. In addition, DDX21 expression promotes PCV2 replication. These results show that DDX21 interacts directly with the PCV2 Cap NLS and the interaction is critical for PCV2 replication.

## Results

### Redistribution of DDX21 is induced by PCV2 infection

**FIGURE 1:**
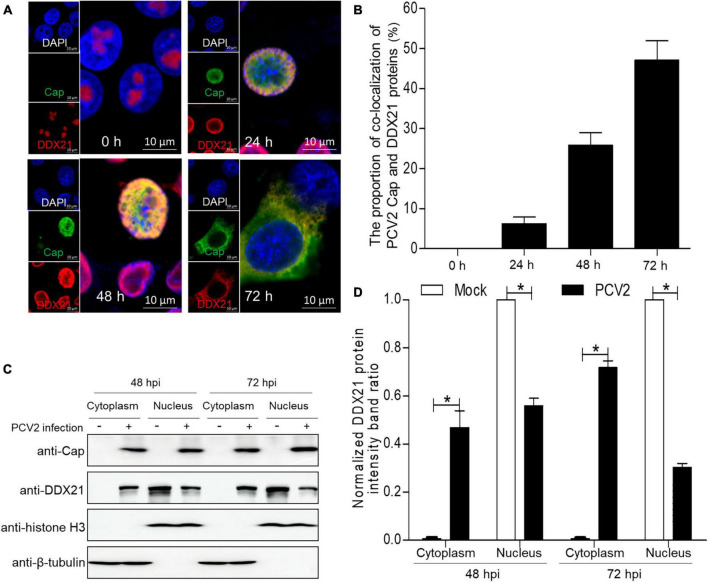
DDX21 relocates from the nucleolus to the cytoplasm induced by PCV2 infection. **(A)** Immunofluorescence analysis of the subcellular localization of DDX21 during PCV2 infection. PK-15 cells were infected with PCV2 at a MOI of 1.0 for 24, 48, 72 h post-infection (hpi). Cells were fixed and stained with mouse mAb against Cap, rabbit anti-DDX21 antibody, FITC-labeled goat anti-mouse IgG, and Alexa Fluor 546-conjugated donkey anti-rabbit IgG. PK-15 cells were observed under a confocal microscope (Supplementary Figure 1). Nuclei were stained with DAPI. Scale bar, 10 μm. **(B)** The proportion of co-localization of PCV2 Cap and DDX21 proteins was analyzed using ImageJ software at 24, 48, and 72 hpi. **(C)** The nuclear and cytoplasmic fractions were extracted after PK-15 cells infected with PCV2 at a MOI of 1.0. At 48 and 72 hpi, the protein samples were prepared and analyzed by western blotting using antibodies against PCV2 Cap and DDX21. Histone H3 and β-tubulin served as fractionation quality controls. **(D)** The DDX21 protein band intensity was analyzed using ImageJ software at 48, 72 hpi. Data are presented as means ± SD of three independent experiments. *p < 0.05.

To demonstrate whether PCV2 infection would induce the subcellular distribution of DDX10, we determined the distribution of DDX21 by confocal microscopy. PK-15 cells were infected with PCV2 at a MOI of 1.0 and fixed at 24, 48, and 72 h post-infection (hpi). The subcellular co-localization of Cap with DDX21 was detected in the nucleus at 24 hpi, and DDX21 was predominantly detected in the nucleolus of mock-infected cells (Figures 1A, B). Likewise, the distribution of DDX21 was changed at 48 hpi, and part of DDX21 redistributed to the nucleoplasm from the nucleolus. The nuclear overlapping of Cap and DDX21 disappeared at 72 hpi, and they were resided in the cytoplasm (Figures 1A, B). In addition, the original and enlarged images of the subcellular distribution of Cap and DDX21 at 72 hpi were showed in Supplementary Figure 1. The nuclear and cytoplasmic fractionation assays were performed to further demonstrate PCV2 infection-induced redistribution. The nuclear and cytoplasmic fractions were isolated at 48 and 72 hpi. The DDX21 was gradually resided in the cytoplasm during PCV2 infection (Figures 1C, D). The results indicate that DDX21 redistributes to the cytoplasm during PCV2 infection.

### DDX21 interacts directly with PCV2 Cap

**FIGURE 2:**
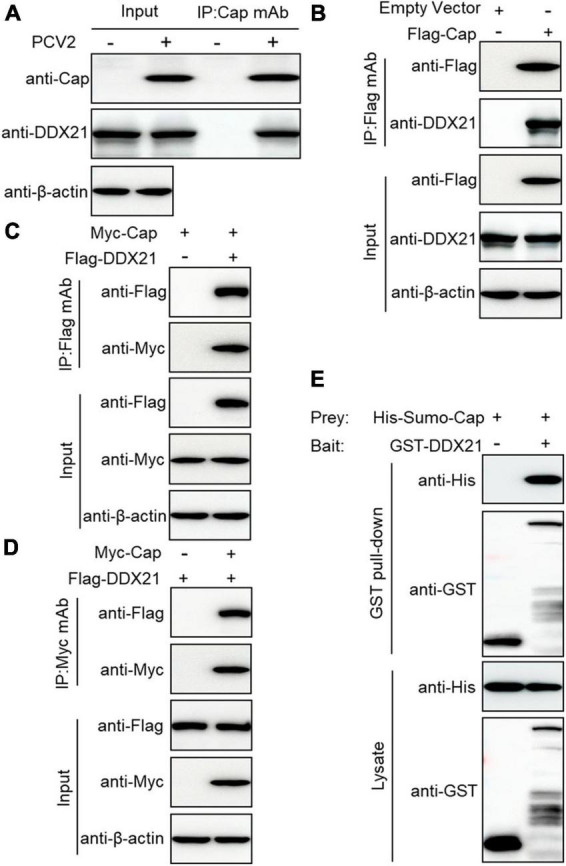
Cap interacts directly with DDX21. **(A)** Immunoprecipitation analysis. PK-15 cells were infected with PCV2 at a MOI of 1.0 for 48 h and cell lysates were immunoprecipitated with purified anti-Cap IgG mAb, followed by immunoblotting with anti-Cap, anti-DDX21, and anti-β-actin antibodies. Besides, the PCV2-infected PK-15 cell lysates were further immunoprecipitated with purified anti-Rep IgG mAb, followed by immunoblotting with anti-Rep, anti-DDX21, and anti-β-actin antibodies (Supplementary Figure 2). **(B)** PK-15 cells were transfected with an empty vector and FLAG-Cap for 48 h. **(C,D)** Co-immunoprecipitation analysis. HEK293T cells were co-transfected with Myc-Cap and FLAG-DDX21 for 48 h, and then the cell lysates were immunoprecipitated with Flag beads **(B,C)** or anti-Myc-purified IgG **(D)**. **(E)** GST pull-down assays. GST or GST-DDX21 proteins were immobilized on GST beads and incubated with His-sumo-Cap. GST or GST-DDX21 proteins in the GST pull-down assays were examined using immunoblotting with anti-His, and anti-GST antibodies, respectively. GST, glutathione-S-transferase.

To investigate the relationship between PCV2 infection-induced DDX21 redistribution, lysates from the PCV2-infected or Cap-transfected PK-15 cells were immunoprecipitated using anti-Cap mAbs or FLAG beads. The results showed that the DDX21 protein interacted with PCV2 Cap (Figures 2A, B). However, viral protein Rep did not interact with DDX21 (Supplementary Figure 2). To investigate the interaction between DDX21 and Cap, HEK293T cells were co-transfected with FLAG-DDX21 and Myc-Cap and used FLAG beads or purified anti-Myc monoclonal antibodies (mAbs) for co-immunoprecipitation. The results revealed that DDX21 interacted with the Cap protein (Figures 2C, D). Additionally, glutathione-*S*-transferase (GST) pull-down assays were performed to investigate whether DDX21 interacts directly with the Cap protein. His-Sumo-Cap and GST or GST-DDX21 were conducted to GST pull-down experiments. The results showed that GST-DDX21 protein interacted with His-Sumo-Cap (Figure 2E). Collectively, these results demonstrate that DDX21 interacts directly with the PCV2 Cap protein.

### NLS of PCV2 Cap is crucial for interaction with DDX21

**FIGURE 3:**
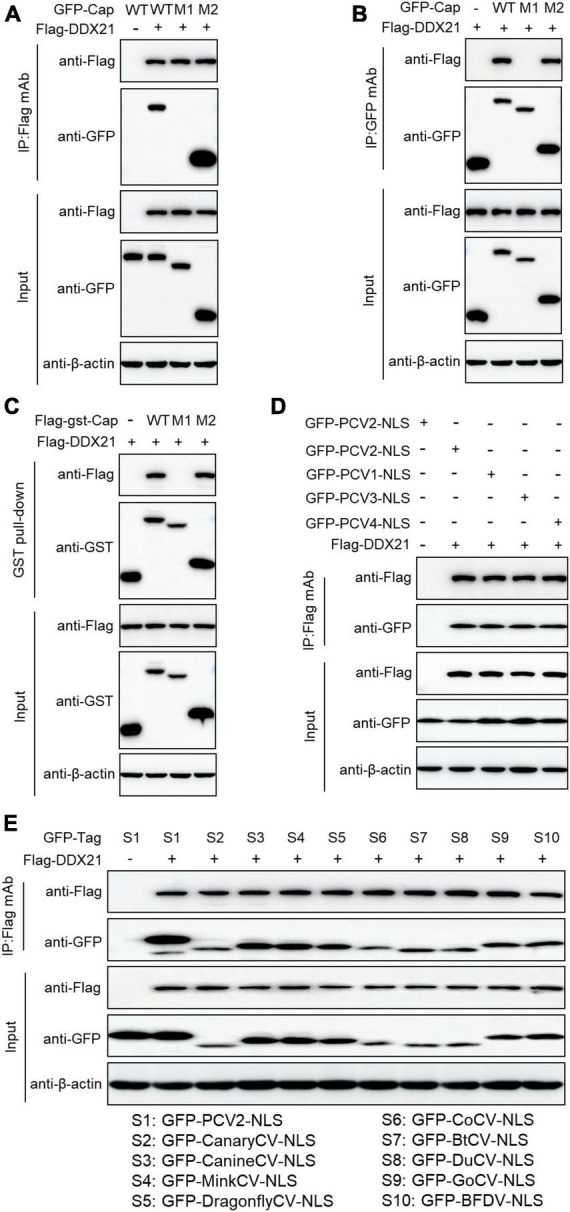
Binding domain identification of Cap with DDX21. HEK293T cells were co-transfected with plasmids containing full-length PCV2 Cap or truncated mutants fused with a GFP-, or FLAG-GST tag, along with FLAG-DDX21 plasmid for 48 h; the cell lysate extracts were immunoprecipitated with Flag beads **(A)** or anti-GFP purified IgG **(B)**, or pulled-down with glutathione S-transferase (GST) beads **(C)** and then detected by western blotting using the indicated antibodies. **(D,E)** The nuclear localization signals (NLSs) within capsid protein of porcine circovirus type 1, 2, 3, 4 and circoviruses from other species were responsible for binding to DDX21. HEK293T cells were cotransfected with plasmids encoding NLSs of PCV1, 2, 3, 4 **(D)** and circoviruses from terrestrial, aquatic and avian species, including pigs, canaries, canines, minks, dragonflies, pigeons, ducks, bats, geese, and parrots **(E)**, along with FLAG-DDX21; cell lysates were subjected to immunoprecipitation and immunoblotting using the indicated antibodies.

To identify the amino acid residues within PCV2 Cap necessary for interaction with DDX21, we transfected the indicated plasmids into HEK293T cells. The results demonstrated that amino acids (aa) of Cap-WT, and Cap-M2 could interact with DDX21, but Cap-M1 did not bind to DDX21 (Figures 3A–C). The results indicate that PCV2 Cap NLS is crucial for binding to DDX21. In addition, Co-IP assays further showed that the NLSs within Cap of all PCVs and circoviruses from other animal species were essential for DDX21 binding as well (Figures 3D, E), which were similar to the previous results (Zhou et al., 2022a), suggesting that Cap binding to DDX21 is very conserved.

### ^763^GSRSNRFQNK^772^ of DDX21 is required for binding to PCV2 Cap

**FIGURE 4:**
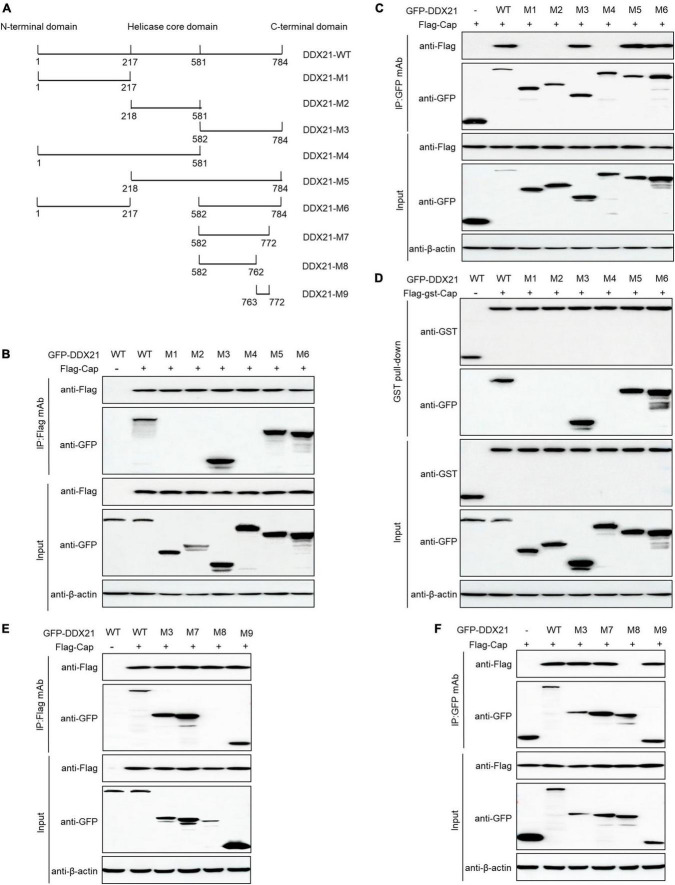
^763^GSRSNRFQNK^772^ of DDX21 is crucial for binding to Cap. **(A)** Schematic representation of the NTD, Helicase D, and CTD of DDX21 and their truncation mutants used in this study. **(B–D)** The DDX21-CTD-(582-784aa) interacted with Cap. HEK293T cells were co-transfected with expression plasmids GFP-DDX21-WT or its serial GFP-DDX21 truncated mutants M1 to M6, together with FLAG-Cap or FLAG-GST-Cap plasmid. The cell lysate extracts were immunoprecipitated or GST pulled-down followed by western blotting using the indicated antibodies. **(E,F)** Identification the critical amino acids of DDX21-CTD essential for interaction with Cap. HEK293T cells were co-transfected with DDX21-WT or DDX21 truncated mutants M3, M7 to M9, along with FLAG-Cap plasmid, and the cell lysate extracts were immunoprecipitated followed by western blotting using the indicated antibodies.

DDX21 comprises an NTD, a conserved helicase domain, and a variable CTD (Fuller-Pace, 2006; Ali, 2021). To identify the amino acid residues essential for DDX21 interaction with PCV2 Cap, we transfected plasmids DDX21-(1-784aa), DDX21-M1-(1-217aa), DDX21-M2-(218-581aa), DDX21-M3-(582-784aa), DDX21-M4-(1-581aa), DDX21-M5-(218-784aa), and DDX21-M6-(1-217aa + 582-784aa) with FLAG-Cap or FLAG-gst-Cap. The results showed that DDX21-WT, DDX21-M3, DDX21-M5, and DDX21-M6 interacted with PCV2 Cap, but DDX21-M1, DDX21-M2, and DDX21-M4 were not able to interact with PCV2 Cap (Figures 4A–D), showing that DDX21-CTD is important for binding to PCV2 Cap.

To identify the amino acids required for interaction with PCV2 Cap within the DDX21-CTD, the online tools were utilized. An unidentified NLS (^763^GSRSNRFQNK^772^) was predicted within the DDX21-CTD. Thus, we co-transfected DDX21-M7-(582-772aa), DDX21-M8-(582-762aa), DDX21-M9-(763-772aa) with FLAG-Cap and subjected them to reciprocal Co-IP assays. The results showed that DDX21-WT, DDX21-M3, DDX21-M7, and DDX21-M9 interacted with FLAG-Cap. However, DDX21-M8 was not able to bind to PCV2 Cap (Figures 4E, F). To sum up, the results demonstrate that ^763^GSRSNRFQNK^772^ of DDX21 is important for binding to PCV2 Cap.

### DDX21 is required for viral progeny production during PCV2 replication

**FIGURE 5:**
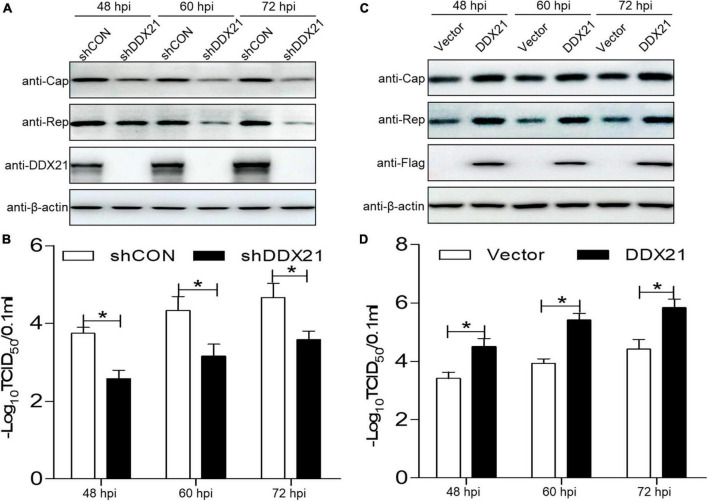
DDX21 promotes the replication of PCV2. **(A,C)** Immunoblotting of the proteins Cap, Rep, DDX21, FLAG, and β-actin in *DDX21*-silenced or DDX21-overexpressing PK-15 cells. The cells were infected with PCV2 at a MOI of 1.0, and shCON-transfected or empty vector-transfected cells served as negative controls. **(B,D)** TCID_50_ values of PCV2 in samples from **(A,C)**. Viral stocks were harvested at 48, 60, 72 hpi. Data are presented as mean ± SD from three independent biological experiments. ns, not significant; *p < 0.05.

To assess the physiological role of DDX21 during PCV2 infection, a short hairpin RNA targeting for *DDX21* and a non-targeted shRNA were delivered to produce cells expressing GFP shRNAs. The *DDX21*-silenced cells were infected with PCV2, and the PCV2 viral proteins Cap and Rep were measured. The PCV2 Cap and Rep protein expression levels were reduced in the *DDX21*-silenced cells compared with those in the shCON (empty vector) controls (Figure 5A). Furthermore, the replicative ability of PCV2 in the *DDX21*-silenced cells was approximately 10-fold lower than that of control cells during infection (*p* < 0.05; Figure 5B). These results suggest that PCV2 replication is reduced in *DDX21*-silenced cells.

As *DDX21* silencing inhibited PCV2 replication, we aimed to determine whether DDX21 overexpression can promote PCV2 replication. PCV2 Cap and Rep expression levels were increased in cells overexpressing DDX21 compared with those in the empty vector controls (Figure 5C). Similarly, PCV2 replication was significantly increased in cells overexpressing DDX21 as well, inferring that DDX21 overexpression promotes PCV2 replication (Figure 5D). In summary, the results indicate that DDX21 expression facilitates PCV2 replication.

## Discussion

In a silent state, DDX21 is located in the nucleolus (Hirai et al., 2013). The subcellular distribution of DDX21 into nucleolus is required for the RNA unwinding (Song et al., 2017). DDX21 regulates viral replication through many ways, including inhibiting viral genome replication and prohibiting virus replication (Watanabe et al., 2009; Abdullah et al., 2021; Li et al., 2022b; Zhao et al., 2022). DDX21 interacts with rRNAs and snoRNAs to facilitate transcription and processing (Flores-Rozas and Hurwitz, 1993). In addition, DDX21 unwinds RNA guanine quadruplexes (Mcrae et al., 2018). Further, DDX21 regulates innate immunity as well. For example, DDX21, DDX1 and DHX36, bind to TRIF to recognize dsRNA (Zhang et al., 2011). DDX21 redistributes from the nucleus to the cytoplasm for inducing innate immune responses during dengue virus infection (Dong et al., 2016). Moreover, DDX21 facilitates redistribution to the cytoplasm to reduce IFN-β production through disrupting the DDX1-DDX21-DHX36 complex during infection (Li et al., 2022a; Zhao et al., 2022). Herein, we demonstrated that DDX21 redistributed to the cytoplasm from the nucleolus during PCV2 infection (Figure 1), suggesting that PCV2 infection induces DDX21 relocation to inhibit innate immunity and facilitate viral replication; however, the underlying mechanisms need further study. We propose that the “dual” location of DDX21 protein plays important functions in RNA metabolism (nucleolus), and regulation of cellular innate immune responses in the cytoplasm. Until now, the study between DDX21 and DNA virus replication was explored only once. Thus, how DDX21 binding to Cap regulates the life cycle of PCV2 remains unclear.

The NLSs of viral proteins are deemed as fundamental elements (Wurm et al., 2001). Certain viral proteins harboring NLSs are important for regulating cell division, and viral replication (Matthews, 2001). There are no consensus sequences within the NLS, despite the sequences being monopartite or bipartite (Silver, 1991). Transcription and translation of the circovirus genome undergoes in the nucleus, and the import of viral DNA requires karyophilic proteins. For PCVs, the NLS is important for virus replication (Cheung and Bolin, 2002). The PCV2 Cap NLS could bind to gC1qR on the nuclear membrane for regulating DNA binding to promote viral replication (Fotso et al., 2016). The results suggest that the PCV2 Cap NLS may promote DNA binding for regulating viral replication as well.

The present results demonstrated that PCV2 Cap binds directly to DDX21 (Figure 2), but we are not sure which form of PCV2 Cap (Cap monomer or Cap-60 polymerized) interacting with DDX21, and future studies are planned to further investigate the PCV2 Cap-DDX21 interaction. Moreover, further investigation is also required to elucidate a specific mechanism. Furthermore, the results showed that the PCV2 Cap NLS is essential for interaction with DDX21 (Figure 3). In addition, we proved that ^763^GSRSNRFQNK^772^ is critical for interaction with the PCV2 Cap NLS (Figure 4), which were similar to the previous results (Zhou et al., 2022a). Since the PCV2 Cap NLS acts as a DDX21 binding site, we hypothesize that DDX21 may facilitate nuclear import of PCV2 Cap. The DDX21-CTD was reported to bind to c-Jun, and its deletion promotes DDX21 redistribution (Holmstrom et al., 2008). Therefore, it is possible that the C-terminus deletion of DDX21 abrogates the interaction with its binding ligand and thus alters its nucleolar distribution. The DDX21 targets the PCV2 Cap to the nucleus via interaction with NLS, thereby facilitating virus replication. PCV2 Cap might enter the nucleus to promote virus replication, or to hijack cell cycle and synthesize cellular proteins during infection (Finsterbusch et al., 2005). In the future, it will be worth demonstrating whether PCV2 Cap interaction with other host proteins regulates virus replication.

In summary, the results of this study show that the PCV2 infection induces translocation of DDX21 to the cytoplasm from the nucleolus. Additionally, we demonstrate that the PCV2 Cap NLS interacts with DDX21. The NLS of the PCV2 Cap and ^763^GSRSNRFQNK^772^ residues at the CTD of DDX21 are necessary for the dual interaction. Furthermore, DDX21 expression facilitates viral replication. Collectively, the results demonstrate that DDX21 interacts directly with the Cap NLS and promotes virus replication, thereby contributing to discovering novel potential targets for prevention and control of PCV2 infection.

## Materials and methods

### Cells and viruses

The PK-15 cell line (CCL-33, ATCC, Manassas, VA, USA) was maintained in minimal essential medium (Gibco, Thermo Fisher Scientific, Waltham, MA, USA). Human embryonic kidney epithelial (HEK) 293T cells (CRL-3216, ATCC) were cultured in Dulbecco’s modified Eagle’s medium (DMEM; Gibco). The *DDX21*-knockdown PK-15 cells were constructed as previously described (Zhou et al., 2022a). All media were supplemented with 10% fetal bovine serum (S711-001S; LONSERA, Shanghai Shuangru Biology Science & Technology Co., Ltd, China) as previously described (Zhou et al., 2022c). PCV2 strain BJW (accession no. AY847748.1) was propagated in PK-15 cells (Liu et al., 2005).

### Antibodies and reagents

Mouse anti-β-actin (M1210-2), anti-histone H3 (R1105-1), anti-glutathione *S*-transferase (GST; M0807-1) mAbs, and rabbit anti-GFP (SR48-02), anti-β-tubulin (0807-2), anti-FLAG (0912-1), and anti-MYC (R1208-1) pAbs were obtained from Huaan Biological Technology (Hangzhou, China). Rabbit mAb against DDX21 (ab182156) was purchased from Abcam (Cambridge, MA). Mouse mAbs to Cap and Rep of PCV2 were produced by our laboratory (Liu et al., 2005). Anti-FLAG affinity resin (A2220) was obtained from Sigma–Aldrich. The ExFect transfection reagent (T101-01/02) was obtained from Vazyme Biotechnology (Nanjing, China). All the antibodies and reagents were purchased and described previously (Zhou et al., 2020, 2022a).

### Plasmid construction and cell transfection

The PCV2 *Cap* plasmids were constructed as described previously (Zhou et al., 2020, 2021a). The resulting plasmids were FLAG-Cap (which the FLAG tag is placed on the N terminal of Cap), MYC-Cap (which the MYC tag is placed on the N terminal of Cap), His-Sumo-Cap (which the His tag is placed on the N terminal of Sumo-Cap), FLAG-GST-Cap, GFP-Cap-WT-(1-233aa), GFP-Cap-M1-(42-233aa), and GFP-Cap-M2-(1-41aa). The full-length and truncated *DDX21* (accession no. KX396051.1) variants were constructed as previously described (Zhou et al., 2022a). The primers used are listed in Table 1. Cell transfection was conducted as previously described (Zhou et al., 2022b).

**TABLE 1:** List of primers adopted in the study.

Gene product	Sense primer (5′ to 3′)	Antisense primer (5′ to 3′)
DDX21-WT-(1-784aa)	ATGCCGGGGAAACTTCGTAGT	TTACTGTCCAAACGCTTTGCT
DDX21-M1-(1-217aa)	ATGCCGGGGAAACTTCGTAGT	CGTCTTTGCTTGTATGGGAAACA
DDX21-M2-(218-581aa)	TTTCACCATGTCTATAGCGGGAA	GATGGCATCTTTACTAGAAGCTTTT
DDX21-M3-(582-784aa)	AGGCTTTTGGATTCTGTGCCT	TTACTGTCCAAACGCTTTGCT
DDX21-M4-(1-581aa)	ATGCCGGGGAAACTTCGTAGT	GATGGCATCTTTACTAGAAGCTTTT
DDX21-M5-(218-784aa)	TTTCACCATGTCTATAGCGGGAA	TTACTGTCCAAACGCTTTGCT
DDX21-M6-(1-217aa + 582-784aa)	ATGCCGGGGAAACTTCGTAGT	AGGCACAGAATCCAAAAGCCTCGTCTTTGCTTGTAT
	ATACAAGCAAAGACGAGGCTTTTGGATTCTGTGCCT	TTACTGTCCAAACGCTTTGCT
	ATGCCGGGGAAACTTCGTAGT	TTACTGTCCAAACGCTTTGCT
DDX21-M7-(582-772aa)	AGGCTTTTGGATTCTGTGCCT	TTTGTTTTGGAATCTGTTGCTT
DDX21-M8-(582-762aa)	AGGCTTTTGGATTCTGTGCCT	ACCTCCTGATCGCTGTCCCCTGAA
DDX21-M9-(763-772aa)	TCGAGGGTAGCAGAAGCAACAGATTCCAAAACAAATAAG	AATTCTTATTTGTTTTGGAATCTGTTGCTTCTGCTACCC

### Confocal microscopy

Confocal microscopy was performed as previously described (Zhou et al., 2022c).

### Sodium dodecyl sulfate-polyacrylamide gel electrophoresis and western blotting

Cells were lysed in lysis buffer after infection or other treatments as for western blotting described previously (Zhou et al., 2020). SDS-PAGE and western blotting were performed as previously described (Zhou et al., 2022a).

### Co-immunoprecipitation and glutathione *S*-transferase pull-down assays

For Co-IP assays, the detailed procedures were conducted as previously described (Zhou et al., 2020). For the conventional and unconventional GST pull-down assays, the detailed procedures were conducted as described elsewhere (Zhou et al., 2021a,b, 2022a).

### Nuclear and cytoplasmic extraction

Nuclear and cytoplasmic components were isolated as previously described (Zhou et al., 2020).

### Statistical analysis

All data are presented as means ± standard deviations (SD). Statistical analysis was conducted using Student’s *t*-test. *p*-values of < 0.05 were considered significant.

## Data availability statement

The original contributions presented in the study are included in the article/Supplementary material, further inquiries can be directed to the corresponding authors.

## Author contributions

JwZ: Conceptualization, Data curation, Investigation, Methodology, Validation, Writing – original draft, Writing – review and editing. JiZ: Data curation, Validation, Writing – review and editing. HS: Conceptualization, Data curation, Methodology, Validation, Writing – original draft. BD: Methodology, Validation, Investigation, Writing – review and editing. NZ: Investigation, Methodology, Validation, Writing – review and editing. QD: Data curation, Investigation, Writing – review and editing. YQ: Validation, Visualization, Writing – review and editing. DW: Formal Analysis, Methodology, Writing – review and editing. YC: Methodology, Writing – review and editing. JG: Data curation, Writing – review and editing. XF: Data curation, Software, Writing – review and editing. LH: Conceptualization, Formal Analysis, Writing – review and editing. JL: Conceptualization, Funding acquisition, Project administration, Resources, Supervision, Writing – original draft, Writing – review and editing.
