# Supplementary figures and images for: DEAD-box RNA helicase 21 interacts with porcine circovirus type 2 Cap protein and facilitates viral replication
Source: Front Microbiol. 2024 Feb 6;15:1298106. doi: 10.3389/fmicb.2024.1298106 (PMC10877017; doi:10.3389/fmicb.2024.1298106)

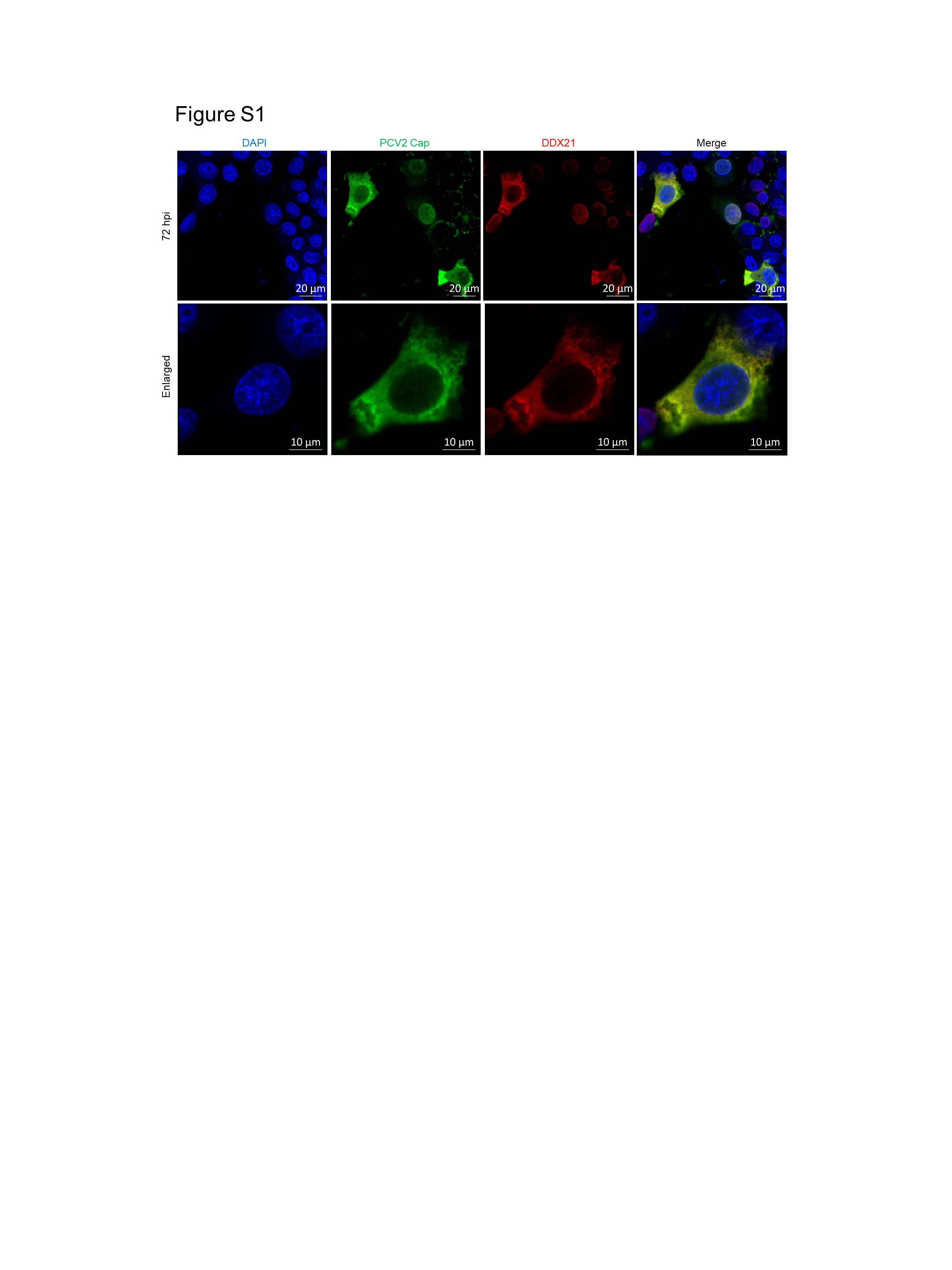

Supplement: Supplementary file 1 [file Image_1.TIF]

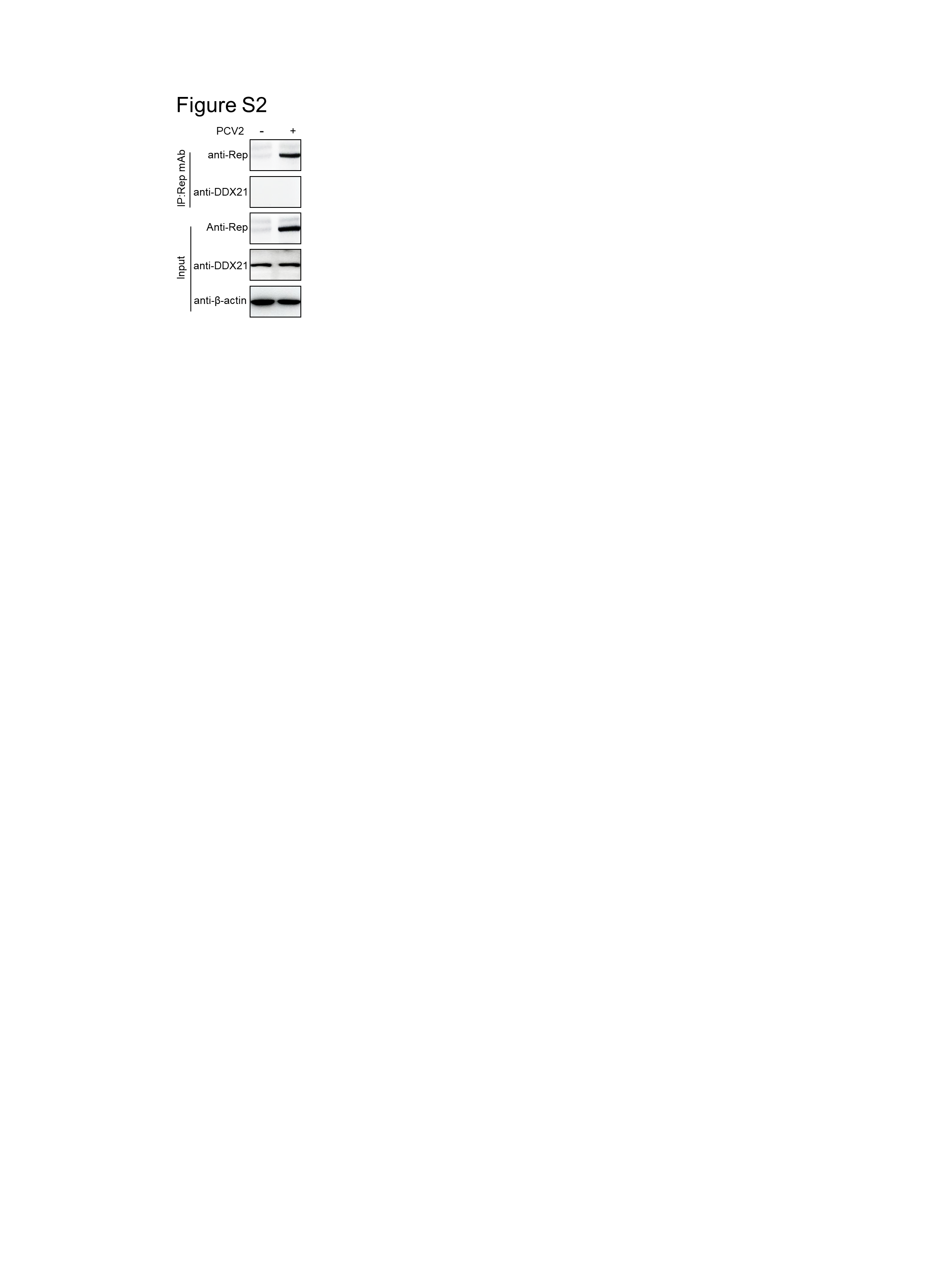

Supplement: Supplementary file 2 [file Image_2.TIF]
